# Arachnoid cysts do not contain cerebrospinal fluid: A comparative chemical analysis of arachnoid cyst fluid and cerebrospinal fluid in adults

**DOI:** 10.1186/1743-8454-7-8

**Published:** 2010-06-10

**Authors:** Magnus Berle, Knut G Wester, Rune J Ulvik, Ann C Kroksveen, Øystein A Haaland, Mahmood Amiry-Moghaddam, Frode S Berven, Christian A Helland

**Affiliations:** 1 Institute of Medicine, University of Bergen, 5021 Bergen, Norway; 2 Institute of Surgical Sciences, Section for Neurosurgery, University of Bergen, 5021 Bergen, Norway; 3 Department of Neurosurgery, Haukeland University Hospital, 5021 Bergen, Norway; 4 Laboratory of Clinical Biochemistry, Haukeland University Hospital, 5021 Bergen, Norway; 5 Department of Mathematics, University of Bergen, 5008 Bergen, Norway; 6 Centre for Molecular Biology and Neuroscience, Institute of Basic Medical Sciences, University of Oslo, Oslo, Norway; 7 Proteomic Unit (PROBE), Department of Biomedicine, University of Bergen, 5021 Bergen, Norway

## Abstract

**Background:**

Arachnoid cyst (AC) fluid has not previously been compared with cerebrospinal fluid (CSF) from the same patient. ACs are commonly referred to as containing "CSF-like fluid". The objective of this study was to characterize AC fluid by clinical chemistry and to compare AC fluid to CSF drawn from the same patient. Such comparative analysis can shed further light on the mechanisms for filling and sustaining of ACs.

**Methods:**

Cyst fluid from 15 adult patients with unilateral temporal AC (9 female, 6 male, age 22-77y) was compared with CSF from the same patients by clinical chemical analysis.

**Results:**

AC fluid and CSF had the same osmolarity. There were no significant differences in the concentrations of sodium, potassium, chloride, calcium, magnesium or glucose. We found significant elevated concentration of phosphate in AC fluid (0.39 versus 0.35 mmol/L in CSF; *p *= 0.02), and significantly reduced concentrations of total protein (0.30 versus 0.41 g/L; *p *= 0.004), of ferritin (7.8 versus 25.5 ug/L; *p *= 0.001) and of lactate dehydrogenase (17.9 versus 35.6 U/L; *p *= 0.002) in AC fluid relative to CSF.

**Conclusions:**

AC fluid is not identical to CSF. The differential composition of AC fluid relative to CSF supports secretion or active transport as the mechanism underlying cyst filling. Oncotic pressure gradients or slit-valves as mechanisms for generating fluid in temporal ACs are not supported by these results.

## Background

Arachnoid cysts (AC) are relatively common benign lesions of the arachnoid, with a reported prevalence as high as 1.1% in the adult population [1]. Clinical presentations of AC include headache, dizziness, seizures [2] and dyscognition [3]. They can be found all along the cranio-spinal axis, but have a marked predisposition for the temporal fossa [4]. The mechanisms underlying the formation and filling of arachnoid cysts are not well understood, but clinical, epidemiological, and laboratory data indicate that genetic mechanisms are involved in the *formation *of arachnoid cysts [5,6]. Three prevailing theories exist for the *filling *of the cyst: 1) active secretion of fluid by cells in the cyst wall [7,8], 2) fluid influx due to an oncotic pressure gradient [9], and 3) trapping of fluid by a valve mechanism [10]. It is conceivable that the chemical composition of the AC fluid relative to the cerebrospinal fluid CSF reflects the mechanism by which the fluid enters the cyst. If the composition is identical to CSF a valve mechanism appears likely, whereas if the filling is caused by oncotic pressure, a higher concentration of proteins in the cyst fluid compared with CSF would be expected. Likewise, cyst fluid composition could reflect the mechanism of transport across the cyst wall, if such a mechanism is involved.

In their study of clinical chemical analysis of cyst fluid in pediatric patients, Sandberg *et al*. [11] described a similar chemical composition to that of reference CSF in the majority of patients investigated, but in 14 of 41 (34%) the protein concentrations were elevated above 0.50 g/L in the cyst fluid. Based on these findings, the authors hypothesized that higher protein content could contribute to the expansion of the cysts by an oncotic pressure gradient. We have recently described the up-regulation of the CSF-secreting cation chloride co-transporter NKCC1 in AC membranes compared with normal arachnoid [8]. This finding supports fluid secretion as the main mechanism of fluid accumulation in AC. The objective of the present study was to analyze the chemical parameters of AC fluid and compare with CSF from the same patient, to gain further knowledge of AC concerning the mechanisms of filling and sustaining of such cysts.

## Methods

### Patients

A total of 15 patients (9 female, 6 male, age 22-77) with unilateral, temporal AC were included. Cyst type and sidedness are summarized in table 1. Patient 3 had previously had a chronic subdural hematoma, most probably caused by the cyst [12]. Patient 4 had undergone previous surgery for the cyst. The other patients had no previous history of intracranial hematomas or surgery. Patients were recruited by written informed consent by the responsible surgeon. This project was approved by The Regional Committee for Medical and Health Research Ethics (REK) of Western Norway (approvals REK 70.03, NSD 9634 and REK 2009/1885).

**Table 1:** Characteristics of patients in study with age, gender, Gallasi-stage [20] and remarks.

Patient	Age (yrs), sex	Side	**Galassi-stage **[20]	Remarks
1	26, f	Left	2	
2	43, m	Left	2	
3	58, f	Left	3	Old hematoma
4	34, f	Left	2	Reoperation
5	22, f	Right	1	
6	36, f	Right	2	Slight hemolysis CSF
7	35, f	Right	2	
8	77, f	Left	1	
9	42, f	Left	1	
10	60, m	Left	2	Slight hemolysis CSF
11	56, m	Right	2	
12	25, m	Left	1	
13	30, f	Left	1	
14	37, m	Left	2	
15	63, m	Left	2	

### Operative technique and fluid sampling

Details for the surgical procedure have previously been given elsewhere [12-14] but a short description is given here. All patients were operated with a craniotomy under general anaesthesia, given as total intravenous anaesthesia (TIVA) with propofol and remifentanyl. Vecuronium bromide (Norcuron^®^) was used as the neuromuscular blocking agent. A burr hole was made with a high-speed drill immediately posterior to the sphenoid wing in order to gain access to the anterior and most basal aspects of the middle cranial fossa. The dura and the underlying cyst membrane were punctured through the burr-hole with a 23 G, 25 mm long syringe connected to an Optidynamic^® ^spinal fluid manometer (Mediplast AB, Malmo, Sweden). After pressure equilibration and registration, a cyst fluid sample (3 - 5 ml) was collected using the manometer tube as a siphon. The sample was immediately transferred to a sterile centrifuge tube for centrifuging and further analytic processing as described below (sample handling). After this procedure, a standard craniotomy with a microsurgical resection and fenestration of the cyst membranes was performed. Before opening the medial cyst wall and thus communicating the cyst interior to the basal arachnoid space/CSF, all cyst fluid was aspirated to avoid cyst fluid contamination of the CSF.

After opening the medial cyst membrane that covered the basal structures (the tentorial slit, the oculomotor nerve, the carotid artery, and the optic nerve), thus creating communication to the basal cisterns and the posterior fossa, a CSF-sample was collected with a pre-cut baby-feeding catheter #6, connected to a 10 ml syringe. The catheter was placed below the tentorium via the tentorial slit and fluid was aspirated gently from the posterior fossa. The collected CSF was transferred to centrifuge tubes and processed in an identical manner to the cyst fluid.

### Sample handling

The samples were transferred to polypropylene tubes (Nunc CryoTube, Thermo-Fischer Scientific, Roskilde, Denmark) and centrifuged for five min at 450 *x g *to remove cells and cell debris. The supernatant was transferred to new polypropylene tubes and immediately stored on dry ice prior to long term storage at -80°C. Such sample handling has previously been demonstrated to reduce degradation of components and cell lysis, which may change the composition of the sample [15].

Samples were thawed for analysis at room temperature and transferred to pre-marked analysis tubes for laboratory analysis.

### Chemical analysis

The samples were analyzed at Laboratory for Clinical Biochemistry, Haukeland University Hospital, 5021, Bergen, Norway. The laboratory is accredited by Norwegian Accreditation (accreditation number "TEST 231") as a testing laboratory and complies with the requirements of NS-EN ISO 15189. Clinical chemistry analysis was performed on a Modular Analytics System by Roche Diagnostics (Roche Diagnostics GmbH, Mannheim, Germany). The analytical coefficient of variation (CV) is noted in parenthesis for each analyte; Sodium (CV 1%), potassium (CV 2%) and chloride (CV 2%), were measured by ion-selective electrode on an ISE 1800 module. Magnesium (CV 2.5%), phosphate (CV 3%), calcium (CV 2%), bilirubin (CV 6%) lactate dehydrogenase (CV 2.5%), protein (CV 1.8%) glucose (CV 2.5%), triglycerides (CV 3%) and iron (CV 2%) were measured by photometic assays on a P 800-module. Ferritin (CV 5%) was measured by an ECLIA (electrochemiluminescence) immunoassay on an E 170-module. Osmolarity (CV 1.5%) was measured by freeze point depression on a Fiske Micro-Osmometer (Fiske Associates, Massachusetts, USA). Immunoglobulins (CV 2-5%) were measured by nephelometry with system specific N antisera to Human Immunoglobulins (Dade Behring Marburg GmbH, Marburg, Germany) on a BN Pro Spec System (Siemens Healthcare Diagnostics, Illinois, USA)

### Statistical analysis

For each of the thirteen patients without previous operations or known injuries to the AC, the differences in concentration between cyst fluid and corresponding CSF were calculated. A paired T-test was then utilized to check if the mean ratio was equal to one. Due to slight hemolysis of two CSF samples (patients 6 and 10), data from these patients were omitted from the statistical analyses for lactate dehydrogenase, ferritin and protein. Two patients (patient 3 and 4) were described clinically as different from the others, and were compared separately with the thirteen native patients using a two-sample t-test assuming equal variances. Correlation between lactate dehydrogenase, ferritin and protein was determined by correlation analysis as a control against a possible contamination of the samples with blood. *P*-values were calculated utilizing Pearson's product moment correlation test. Statistical analysis was performed using the statistics software package R version 2.10.1 (The R foundation for Statistical Computing, Vienna, Austria).

## Results

Chemical analysis of AC fluid and CSF, obtained during elective surgery for arachnoid cysts from 15 patients, was performed in a routine hospital laboratory. The results from the measurements are presented in table 2. There was no significance difference in osmolarity or concentrations of sodium, potassium, chloride, calcium, magnesium or glucose between AC fluid and CSF. The concentration of phosphate was higher in AC fluid relative to CSF (0.39 versus 0.35 mmol/L; *p *= 0.02), while the concentration of total protein (0.30 versus 0.41 g/L; *p *= 0.004), lactate dehydrogenase (17.9 versus 35.6 U/L; *p *= 0.002) and ferritin (7.8 versus 25.5 ug/L; *p *= 0.001) was significantly lower in AC relative to CSF. Bilirubin, iron, triglycerides and immunoglobulins were below quantification limit for analysis setup. Patient 12 was identified as an extreme outlier for protein measurement and was excluded from this analysis.

**Table 2:** Results of chemical analysis of AC fluid and CSF in the same patients, with units, number of samples in calculation (n), mean results for AC fluid and CSF with standard error of mean (SEM) and means of AC fluid/CSF ratio.

	Unit	n	Mean AC fluid +/- SEM	Mean CSF +/- SEM	Mean cyst/CSF
Sodium	mmol/L	13	142.23 +/- 2.14	142.08 +/- 2.71	1.00
Potassium	mmol/L	13	2.47 +/- 0.04	2.35 +/- 0.07	1.05
Chloride	mmol/L	13	121.23 +/- 1.94	120.15 +/- 2.54	1.01
Calcium	mmol/L	13	1.07 +/- 0.01	1.03 +/- 0.03	1.04
Magnesium	mmol/L	13	1.20 +/-0.01	1.14 +/- 0.03	1.05
Phosphate	mmol/L	13	0.39 +/-0.01	0.35 +/- 0.01	1.11*
Glucose	mmol/L	13	2.85 +/- 0.09	3.13 +/- 0.11	0.92
Protein	g/L	10	0.30 +/-0.03	0.41 +/- 0.04	0.71**
Lactate dehydrogenase	U/L	11	17.91 +/- 2.93	35.55 +/- 4.61	0.57**
Ferritin	ug/L	11	7.82 +/- 1.00	25.55 +/- 6.20	0.31**
Osmolarity	mosmol/L	13	290.15 +/-1.07	290.08 +/- 0.96	1.00
					
IgG	g/L		< 1.50	< 1.50	
IgA	g/L		< 0.25	< 0.25	
IgM	g/L		< 0.18	< 0.18	
Iron	umol/L		low	low	
Triglycerides	mmol/L		low	low	

Significance levels are denoted as *p *< 0.05 (*) and *p *< 0.01 (**). Data from patients #3 and #4 are excluded as clinical outliers from this table. Data from patients #6 and #10 were excluded for protein, lactate dehydrogenase and ferritin due to slight hemolysis in the CSF. Patient #12 was excluded from protein concentration measurement as an extreme statistical outlier.

Correlations were calculated between possible blood contamination parameters ferritin, lactate dehydrogenase and protein on the CSF samples without observed blood contamination (n = 11). The correlation between ferritin and LD was 0.77 (*p *= 0.006), ferritin and protein was 0.36 (*p *= 0.312) and lactate dehydrogenase and protein was 0.34 (*p *= 0.34). The protein concentrations in the cyst fluid from the two clinically different patients (patient 3 and 4, respectively 6.16 g/L (hematoma) and 3.54 g/L (previous operation)) were significantly elevated relative to the others (*p *< 10^-16^).

## Discussion

The aim of the present study was to collect information that may contribute to the understanding of the mechanism for arachnoid cyst filling. If the cyst were filled by a simple valve mechanism, which has been observed in suprasellar cysts, it would be expected that the composition of the cyst fluid would be identical to that of CSF. On the other hand, if the filling was caused by oncotic pressure, one would expect a difference in osmolarity between the AC fluid and CSF as well as a higher protein content in the AC. If the underlying mechanism were active secretion or transport, this would probably cause a different concentration of certain molecules or ions depending on the underlying transport mechanisms. We found an isotonic AC fluid with a lower protein concentration than in CSF; this is not consistent with an oncotic pressure filling mechanism.

Macromolecules such as albumin (67 kDa), ferritin (24 subunits between 19 and 21 kDa, total weight around 450 kDa) and lactate dehydrogenase (tetramer of about 37 kDa, total weight around 140 kDa) would be expected to pass freely through a slit in the cyst membrane. However, the ratio between cyst fluid and CSF for these three protein complexes was reduced (dependent on size), from 0.73 for protein (of which 2/3 albumin) to 0.31 for ferritin. Our findings are therefore contradictive of a slit valve mechanism underlying the filling of temporal AC.

The skewed distribution of phosphate could imply a selective or active transport of fluid and solutes over the AC membranes. This is consistent with previous findings of morphological and enzyme ultracytochemical structures in the wall of arachnoid cysts assumed to be capable of fluid secretion, as reported by Go *et al *[7]. Furthermore, our group has recently published evidence that there are differences between arachnoid cysts and normal arachnoid tissue in that the Na-K-Cl cotransporter NKCC1 is up-regulated in arachnoid cysts compared with normal arachnoid [8], and a small subset genes are differentially expressed in arachnoid cysts compared with normal arachnoid tissue [5]. The phosphate level in the CSF is kept lower than in the blood [16], due to active transport mechanisms in the choroid plexus epithelia. Higher phosphate concentration in the cyst fluid could imply that the cyst epithelium is either not, or is differentially equipped with transport mechanisms relative to the choroid plexus. There are several lines of evidence suggesting that co-transporters such as GLUT1, MCT1 and NKCC1 have the ability to transport water along with their respective substrates, regardless of the osmotic gradients [17].

As we have only studied temporal cysts, we cannot generalize the assumption of an active transport mechanism to all ACs. For other locations, other fillings mechanisms may well exist, such as a slit-valve in supracellar cysts [18,19]. In our study, two patients differed from the others - one with a previous hematoma in close proximity to the cyst and one being a reoperation. The protein levels in the cyst fluid from these patients were significantly higher than in the rest of the study group. In their study Sandberg *et al *[11] found that 14 of the 41 patients had markedly higher protein concentrations (above 0.5 g/L) in the cysts; four of those had extreme values such as the ones we observed. The cause for some cysts to have a marked elevated protein level is not well understood, but products from previous bleeding in the cyst may be one explanation. Correlation analysis between protein, ferritin and lactate dehydrogenase was performed as a statistical control to rule out blood contamination as a possible explanation of the protein content in the CSF. The protein concentration of CSF relative to plasma is about 0.5-1%; a leak of plasma to the CSF would introduce a large source of error to the analysis. If the difference in concentration of protein, lactate dehydrogenase and ferritin was caused by blood contamination, the correlation should be expected to be significant and close to 100%. These correlation results do not show a strong association between assumed blood contaminants and the findings, thus not supporting blood contamination as explaining variable.

A limitation for this study is the small number of samples. Applied on such a dataset, the T-test is vulnerable to outliers such as the excluded patient 12 in protein concentration (outlier plots not shown). Nonetheless, our results can support reflections around the filling mechanisms for arachnoid cysts. As far as the authors know, this is the first publication of AC fluid analysis matched with CSF from the same patient.

## Conclusions

The chemical composition of AC fluid found in this study does not support an oncotic pressure or valve mechanism as responsible for filling an AC. Due to the pattern of differences, we postulate that the filling mechanism for temporal AC is by either a selective or active transport mechanism or a secretion from the cyst-lining cells.

## Competing interests

The authors report no conflict of interest concerning the materials or methods used in this study or the findings specified in this paper.

## Authors' contributions

MB applied for ethical approval, collected the samples, processed the samples for analysis, processed the data and drafted and edited the manuscript. KGW is the senior neurosurgeon and operated on the patients, devised sampling technique and drafted and edited parts of the manuscript. RJU applied for ethical approval, organized clinical chemical analysis and participated in editing of the manuscript. ACK worked on the biobanking design of the study, discussion around and editing of the manuscript. OAH did the statistical analysis and statistics interpretation. MAM contributed on writing the discussion part and the intellectual interpretation of the data. FSB worked on biobanking design and the editing of the manuscript. CAH conceived of this study, operated on the patients and helped draft and edit the manuscript. All authors read and approved the final manuscript.
